# Supplementary material for: In situ visualization of bacterial populations in coral tissues: pitfalls and solutions
Source: PeerJ. 2016 Sep 20;4:e2424. doi: 10.7717/peerj.2424 (PMC5036075; doi:10.7717/peerj.2424)
Supplement: Table S1 — Identified pitfalls and potential solutions to overcome challenges of applying FISH approaches to coral tissues. [file peerj-04-2424-s002.docx]

**Supplementary Table 1**. Identified pitfalls and potential solutions to overcome challenges of applying FISH approaches to coral tissues

| **Pitfall:** Coral tissue and *Symbiodinium* autofluorescence emission spectra often overlap with FISH probe fluorochrome emission spectra. This overlap confounds effective differentiation between probe signal and background autofluorescence. | **Solution:** Careful selection of FISH probe fluorochromes is essential to minimise overlap between the emission ranges of probes and coral and *Symbiodinium* autofluorescence. A strong understanding of coral and *Symbiodinium* autofluorescence emission spectra is therefore required.  Use of advanced imaging hardware, such as laser confocal scanning microscopes (LSCM), can significantly improve detection of probe signal over background autoflorescence and are highly recommended when available. | **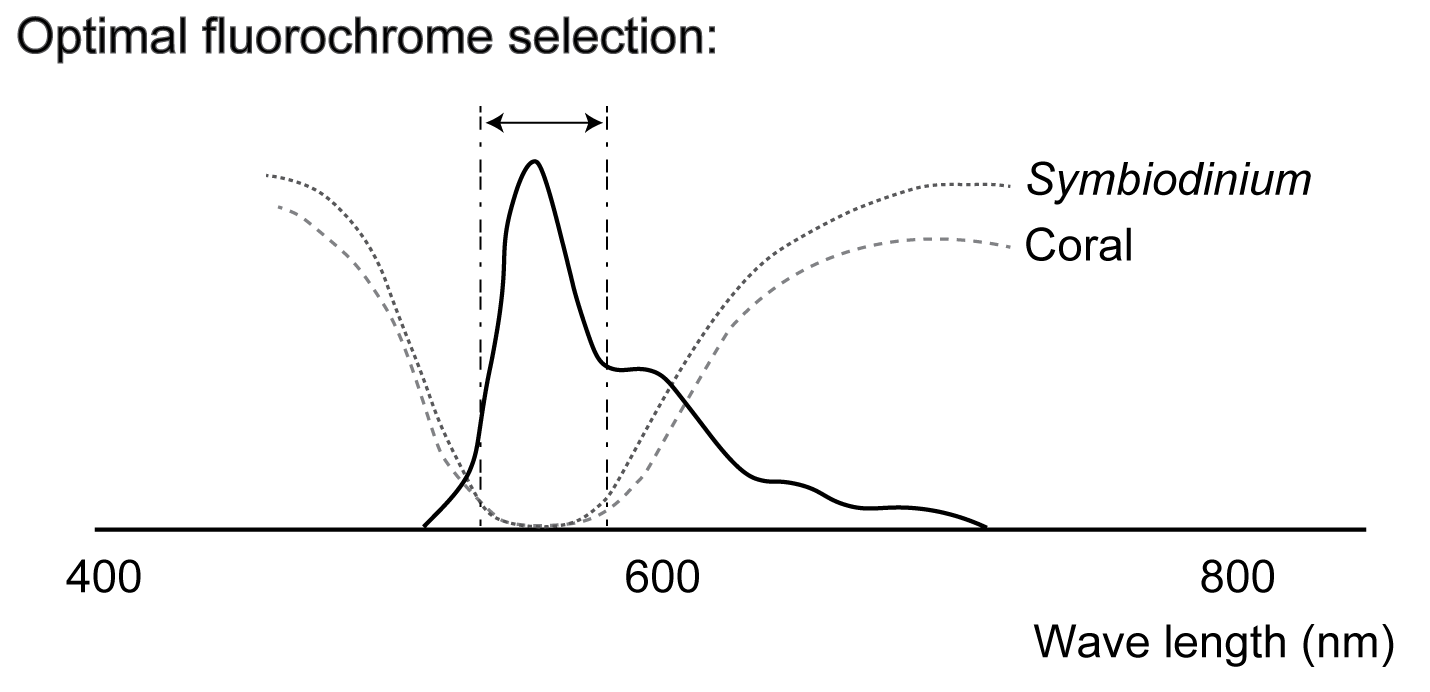** |
| --- | --- | --- |
| **Pitfall:** Coral cellular structures, including nematocysts, spirocysts and granular gland cells, bind non-specifically to FISH probes and are thus difficult to distinguish from true positive detection. | **Solution:** Familiarity with coral cellular structures is essential to distinguish non-specific fluorescent cell types from specific bacterial hybridisation based on morphological characteristics. | **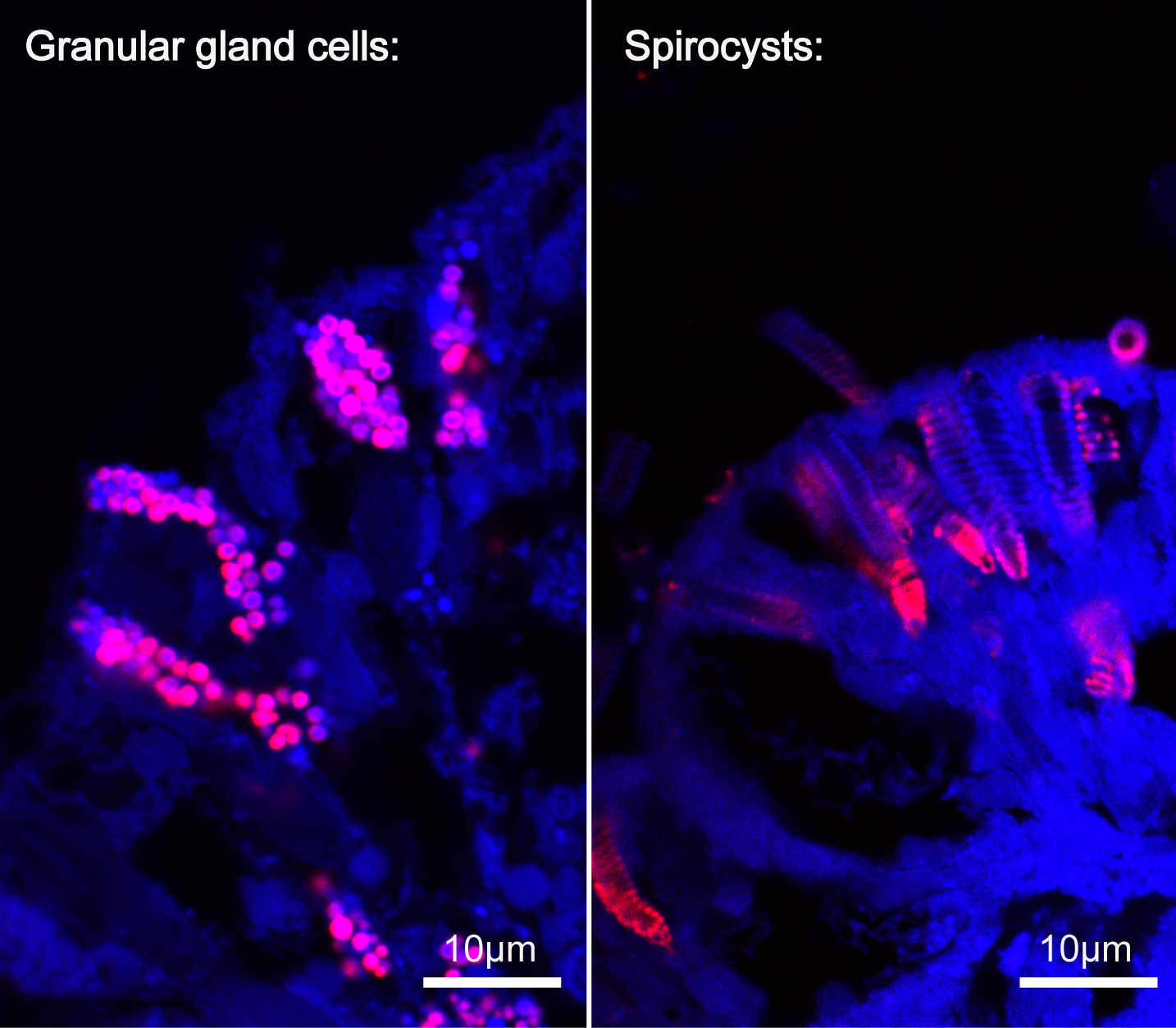** |
| **Pitfall:** Low fluorescent signal due to low copy number of target rRNA molecules can make it difficult to visualise bacterial cells in coral tissue sections. | **Solution:** Florescent signal amplification techniques such as CARD-FISH can increase signal intensity when target rRNA copy numbers are low. These approaches have proven successful in coral FISH studies (see Bayer et al 2013, Neave et al 2016). | 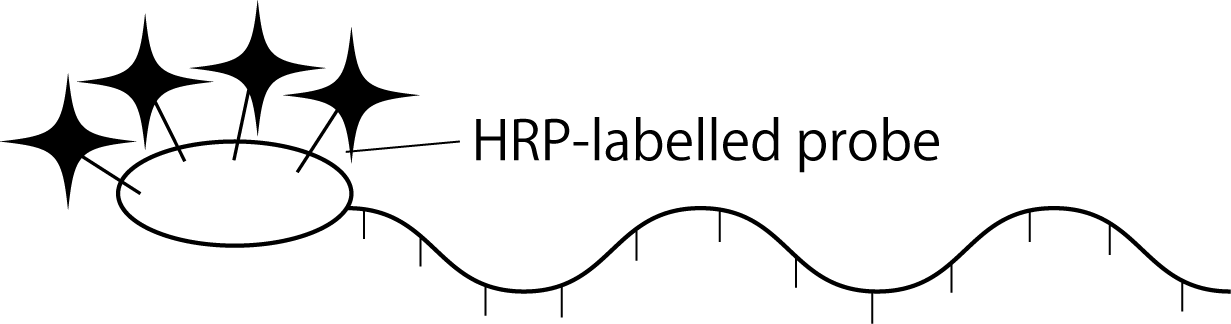 |
| **Pitfall:** The use of nonsense probes to detect non-specific binding can be problematic as the nematocysts, spirocysts and granular gland cells bind the probe. During a 2-step process of hybridising with a non-sense probe labelled with one flourochrome and then binding with another specific target probe labelled with a different flourochrome, these cellular structures hybridise to both probes. | **Solution:** Nonsense probes should still always be applied to tissue sections to detect and diagnose non-specific probe binding. However, familiarity with coral cellular structures is essential to distinguish these non-specific fluorescent cell types from targeted probe signals. | 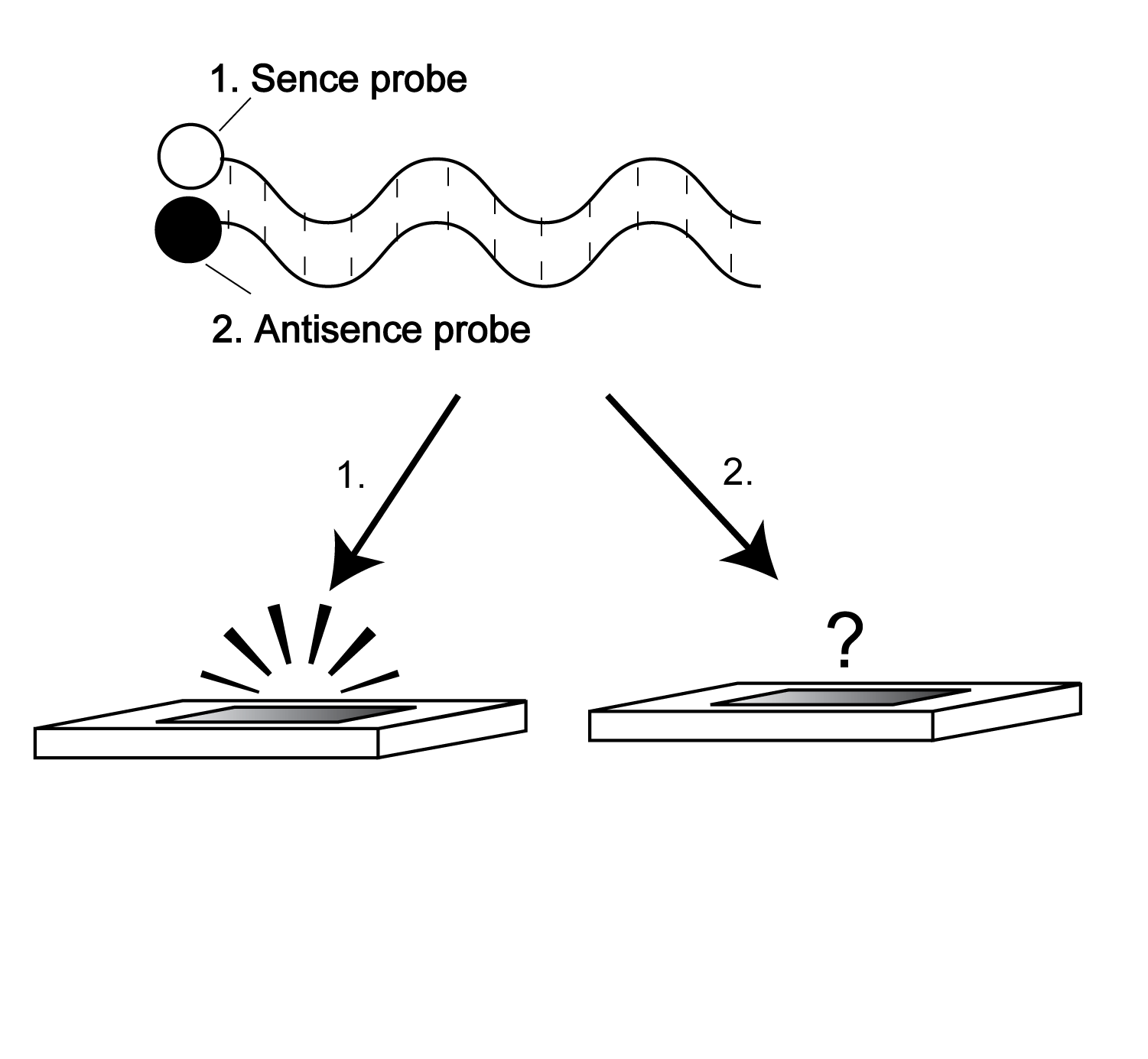 |
| **Pitfall:** While the use of advanced imaging hardware (such as a LSCM) combined with florescent signal amplification techniques (such as CARD-FISH) is recommended, such approaches have the potential to confound the specific detection of bacteria in coral tissues. For example, the signal intensity of non-specific binding to spirocysts and granular gland cells is often much greater than that of specific probe binding to bacteria. This property allows a distinct colour to be assigned to these false positives so that they can be disregarded. The application of CARD-FISH can intensify true positive signals to a level comparable to those associated with non-specific binding making intensity-based differentiation difficult. | **Solution:** Care must be exercised when using florescent signal amplification techniques in combination with advanced imaging hardware that allows spectral profiling at the pixel scale. Again, familiarity with coral cellular structures is essential to  ensure all detected signals are from bacteria specific targets and not background non-specific cellular structures. | **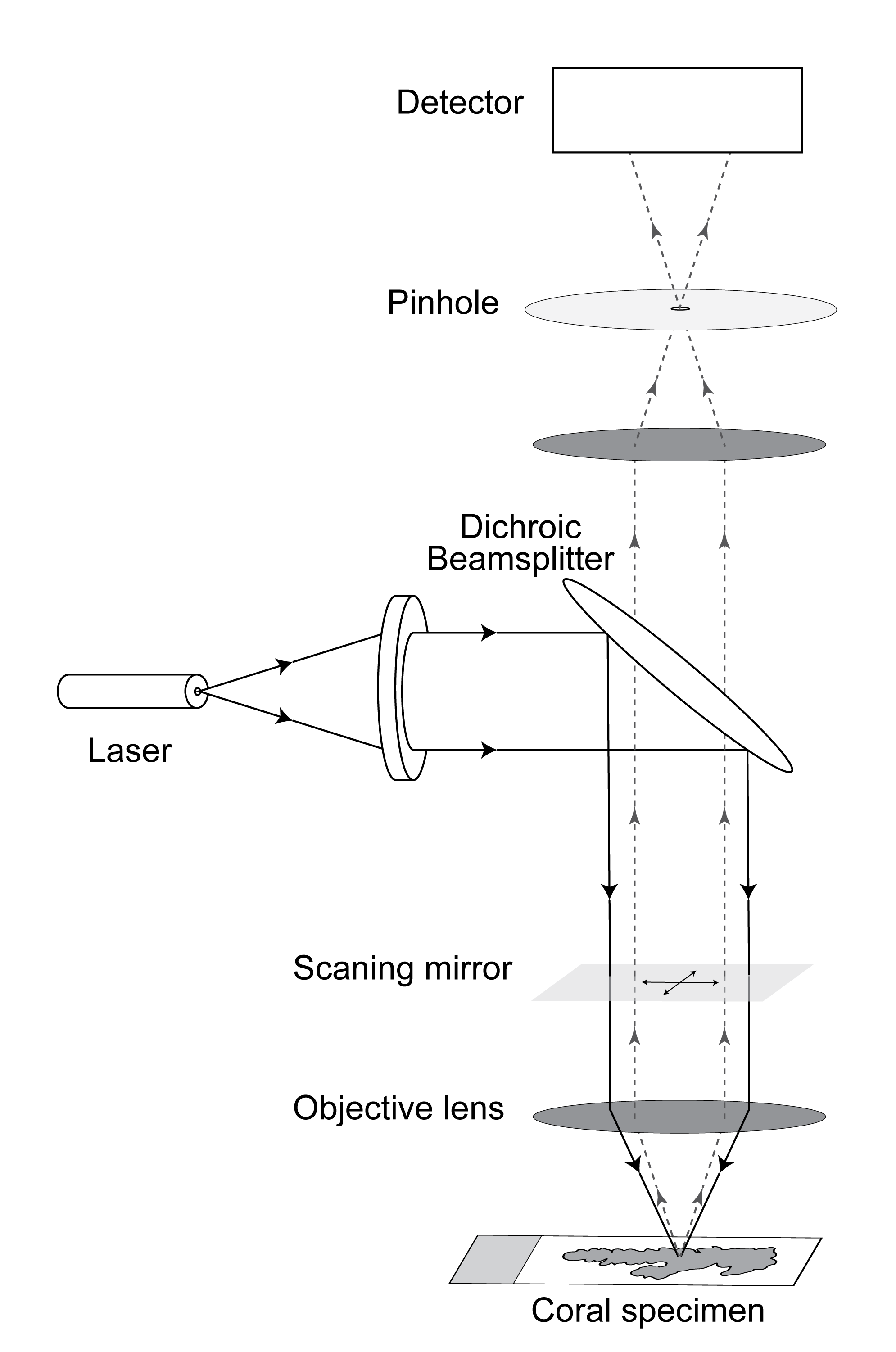** |
